# Role of pyruvate dehydrogenase complex in traumatic brain injury and Measurement of pyruvate dehydrogenase enzyme by dipstick test

**DOI:** 10.4103/0974-2700.50739

**Published:** 2009

**Authors:** Pushpa Sharma, Brandi Benford, Zhao Z Li, Geoffrey SF Ling

**Affiliations:** 1 Department of Anesthesiology, Uniformed Services University of the Health Sciences, Bethesda MD 20814, USA; 2 Department of Neurology, Uniformed Services University of the Health Sciences, Bethesda MD 20814, USA; 3 Department of Biomedical Instrumentation Center, Uniformed Services University of the Health Sciences, Bethesda MD 20814, USA

**Keywords:** Brain, dipstick, mitochondria fluid percussion injury, pyruvate, pyruvate dehydrogenase

## Abstract

**Objectives:**

The present study was designed to investigate the role of a mitochondrial enzyme pyruvate dehydrogenase (PDH) on the severity of brain injury, and the effects of pyruvate treatment in rats with traumatic brain injury (TBI).

**Methods:**

We examined rats subjected to closed head injury using a fluid percussion device, and treated with sodium pyruvate (antioxidant and substrate for PDH enzyme). At 72 h post injury, blood was analyzed for blood gases, acid-base status, total PDH enzyme using a dipstick test and malondialdehyde (MDA) levels as a marker of oxidative stress. Brain homogenates from right hippocampus (injured area) were analyzed for PDH content, and immunostained hippocampus sections were used to determine the severity of gliosis and PDH E1-∞ subunit.

**Results:**

Our data demonstrate that TBI causes a significant reduction in PDH enzyme, disrupt-acid-base balance and increase oxidative stress in blood. Also, lower PDH enzyme in blood is related to the increased gliosis and loss of its PDH E1-∞ subunit PDH in brain tissue, and these effects of TBI were prevented by pyruvate treatment.

**Conclusion:**

Lower PDH enzyme levels in blood are related to the global oxidative stress, increased gliosis in brain, and severity of brain injury following TBI. These effects can be prevented by pyruvate through the protection of PDH enzyme and its subunit E-1.

## INTRODUCTION

Traumatic brain injury (TBI) is the predominant cause of death and disability under 44 years and above 65 years of age.[1] Despite the major improvements in the acute care of head injury victims, no effective pharmaceutical strategies exist for preventing the secondary injury cascade, oxidative stress and metabolic dysfunctions that occurs after brain injury.[2,3] The biochemical analysis, molecular biology and electron microscopic studies of post-mortem human brain with TBI emphasized the role of mitochondrial dysfunctions in deficient cellular energy metabolism and increased neuronal cell death.[4]

Traumatic brain injury results in increased oxidative stress that can oxidize mitochondrial proteins – responsible for the production of cellular energy in the form of adenosine triphosphate (ATP). Among the key mitochondrial metabolic enzymes, PDH is very sensitive to oxidative stress.[5] This enzyme is especially important to the brain that relies mainly on carbohydrate metabolism. In injured brain cells, PDH is believed to be inactive and therefore contributes to the hyperglycemia and lactic acidosis due to reduced glucose metabolism.[6,7] Pyruvate has been widely recognized as a metabolic substrate with multiple antioxidant properties and a natural allosteric stimulator of PDH, but its effects on TBI are not known.[8–11]

In this study, we have examined the consequences of preventing the loss of total PDH enzyme by pyruvate treatment on the severity of brain injury in a rat model of lateral fluid percussion injury. One of the important features of this study is the comparison of total PDH enzyme in blood and brain tissue using a dipstick assay. Our principle behind the use of total blood PDH contents as a biomarker of TBI is that the dysfunctional mitochondria in injured brain results in leakage of toxic free radicals into its matrix, cytosol, and subsequently into the circulation resulting in secondary cell injury and neuronal cell death.[3,12,13] If this is true, then the consequences of TBI on oxidative stress should reflect the PDH content/activity in blood because peripheral blood mononuclear cells (PBMC) also contains mitochondria. In addition, hyperglycemia and increased lactic acidosis in blood are commonly related to the neurological dysfunctions. However, the relationship between these events, severity of brain injury and PDH enzyme has not been established. Therefore, the discovery of tools/techniques to determine the total PDH /PDH activity in blood can provide a new direction in the diagnosis, prevention and treatment of altered cellular metabolism in injured brain.

### Settings and design

The present study investigates 1) the effects of pyruvate on TBI in a rat model of fluid percussion type of brain injury, and 2) whether total PDH enzyme in blood can be a suitable biomarker of TBI. Using a blood-based dipstick test for PDH enzyme, we have examined the relationship between blood PDH levels, serum biochemical parameters, oxidative stress and ultra-structure of brain in rats subjected to lateral fluid percussion injury and treated with pyruvate.

## MATERIALS AND METHODS

Adult, male Sprague–Dawley rats (325–375 g) were individually housed in an environmentally controlled atmosphere under a 12-h light–dark cycle. Food and water were available ad libitum. Rats were allowed for one week to acclimate to the vivarium before use. All procedures confirmed to NIH guidelines and were approved by the Institutional Animal Care and Use Committee of our Uniformed Services University of the Health Sciences, Bethesda, Maryland.

### Induction of traumatic brain injury

Traumatic brain injury was induced in rats using a fluid percussion injury device (VCU Biomedical Engineering, Richmond, VA, USA). The procedure was initially described by McIntosh *et al*. and modified by Ling *et al*.[3,12,13] The fluid percussion device is a Plexiglas tube 60 cm long, 4.5 cm in diameter and filled with distilled water. The tube is sealed with a piston on one end, and a male Luer-loc syringe and pressure transducer on the other end. Rats were positioned in a rodent stereotaxic instrument (Kopf) under continuous anesthesia (2–2.5% isoflurane and remaining oxygen through a fitted nose cone). After exposing the skull by midline incision, a 5-mm craniotomy was conducted 3 mm posterior to bregma and 2 mm lateral to midline over the right cerebral hemisphere. Then, a female Luer-Lock fitting cap was cemented to the cranium. At the time of injury, the cap was connected to a male Luer-Lock fitting, which is the terminator of a tube connected to a Plexiglas cylindrical reservoir filled with sterile saline. Upon impact, a metal pendulum struck the piston of the reservoir from a predetermined height, and generated a 22 ms intracranial fluid pulse of 2.5 atm that briefly displaced the dura and underlying neural tissue. After injury, the Luer-Lock fitting cap was removed, the incisions sutured and the animals were allowed to recover. A warming pad was used to maintain the animal's rectal temperature at 37°C throughout the surgical procedure.

### Animal groups and treatment

Twenty-seven rats were randomly assigned to one of the three groups (9 animals per group). Group 1 was a sham-surgery control group that underwent all of the surgical procedures but did not receive brain injury. Group 2 received lateral fluid percussion TBI. In this group, pyruvate treatment was replaced with 0.9% sterile saline as vehicle. Animals in group 3 received sodium pyruvate (1 g/kg, intraperitoneal injection) within 15 min after surgery and then every 24 h. At 72h, after TBI or sham treatment, rats were euthanized by administering the higher doses of isoflurane.

### Collection and analysis of blood and brain tissue samples

Transcardial blood samples (0.5 ml) were taken from anesthetized animals using a heparinized syringe (100 U/ml, heparin in 0.9% saline) from the left ventricle. 50 µl blood was used immediately for PDH dipstick assay. The remaining blood was centrifuged for 5 min at 10,000 revolutions per minute to obtain serum. Base excess, pH and blood gases were determined by Ultra Stat Blood gas analyzer (IL 1610, Instrumentation Laboratories, Lexington, KY). Serum lactate and pyruvate were measured by CMA 600 (Microdialysis Analyzer). In brief, the analyzer enzymatically converts lactate and pyruvate to hydrogen peroxidase in the presence of 15% lactate oxidase, which catalyses a reaction between H_2_O_2_ and other substrates in the presence of NAD (for lactate reaction) or NADH (for pyruvate reaction) to form the red-violet-colored quinine diimine. The rate of formation of the quinine diimine is measured at 546 nm, and is proportional to the lactate, pyruvate contents. The calibration standards contained serial dilutions of 10-mM L-lactate stock solution and pyruvate in serial dilutions from 2-mM stock solution in double-distilled deionized water. The ratio of serum lactate/pyruvate content was calculated in duplicate for each sample.

For dipstick test, brains from deeply anesthetized animals (*n* = 6 per group) were removed, and immediately dipped in liquid nitrogen, pulverized and stored at –80°C. Remaining three animals from each group were transcardially perfused with 4% PBS buffered paraformaldehyde. Fixed brains were cryoprotected and stored in tightly-capped tubes at –80°C until sectioning. Frozen coronal sections of hippocampus were used for immunohistochemistry.

### Dipstick test to determine total PDH enzyme in blood and brain

The PDH complex from blood or tissue lysate (right hippocampus) was immunocaptured on the dipstick (Mitosciences) according to the manufacturer's guidelines. In brief, detector PDH antibodies, conjugated to gold, are dried in the wells of a 96-well plate. Either 20 µl whole blood (fresh) or brain tissue lysates from the hippocampus (20µg protein) were added to each well in duplicate. The dipstick was inserted in wells containing samples, and within 10 minutes the line for each target was revealed as the protein-detector secondary antibody-gold complex binds with the capture antibodies. A positive control goat anti-mouse antibody line (top band in Figure 2) is included on all assays to ensure that adequate wicking of the sample occurred. This intensity of color band on the dipstick was measured using a flat bed scanner and NIH image software. All determinations were performed in duplicate.

### Measurement of lipid peroxidation

Serum was used for the measurement of malondialdehyde (MDA) contents using a colorimetric thiobarbituric acid assay kit from Calbiochem, La Jolla, CA. The MDA levels were expressed as nmol/ml serum.

### Protein assay

Protein contents in right hippocampal tissue homogenates were determined according to Bradford assay using bovine serum albumin as standard.[17]

### Immunohistochemistry

The right hippocampus was sectioned (25 *µ*m) using a Leitz cryostat microtome and sections were collected in buffered 4% paraformaldehyde (pH. 7.4). For double labeled immunostaining, primary monoclonal mouse anti-glial fibrillary acidic protein (GFAP; Sigma, St. Louis, MO 1:500) and polyclonal mouse anti-PDH E1-∞ (1:500, Mitosciences) were used. After primary antibody incubation, sections were washed three times for 5 min with PBS and then incubated in rhodamine or fluorescine-conjugated secondary antibodies for 30 min at room temperature (Jackson Immunochemicals, West Grove, PA, 1:500). All slides were washed 3 × 5 min, and then mounted in Vectashield with 4', 6-diamidino-2-phenyindole (DAPI), coverslipped, sealed with nail polish and stored at 4°C. The sections were visualized under Nikon inverted-stage microscope (25× and 100× magnification). Digital images were captured with a SPOT microscope camera (Diagnostic Instruments, Sterling Heights, MI).

### Statistical analysis used

Between groups analyses of means were accomplished by one way analysis of variance followed by post-hoc Tukey's honestly significant difference (HSD). Dunnett's test was used for multiple comparisons against sham. Values were considered statistically different when *P* < 0.05.

## RESULTS

### Pyruvate effects on acid-base parameters in blood after TBI

Table 1 shows that there is no significant change in serum pH throughout the protocol. In contrast, TBI caused significant increases in lactate and lactate to pyruvate ratios, and decreases in base excess as a sign of metabolic acidosis. Although, administration of sodium pyruvate did not reduce serum lactate levels, but significantly reduced the lactate to pyruvate ratio, the more relevant indicator of metabolic stress. TBI also resulted in a rapid increase in serum glucose levels in vehicle treated group, which was corrected by sodium pyruvate. These observations are in accordance with the several published papers on brain injury and serum markers of metabolic dysfunctions, and thus confirm the validity of our brain injury rat model.

**Table 1:** The hemodynamic and laboratory measurements were made immediately after blood collection at 72-h post injury period

Parameter	Sham (9)	TBI (9)	TBI + Pyruvate (9)
pH	7.4±0.1	7.3±0.4	7.5±0.3
SO_2_%	83.5 ±3.7	60.4±1.5*	99.90±6.2*^#^
Glucose (mg/dL)	149±5.2	209±4.1*	173±6.3*^#^
Lactate (mmol/L)	1.4±0.2	3.2± 0.3*	6.1±0.5*^#^
L/P ratio	9.0±0.9	22.8±3.5*	11.5±1.6^#^
BE-ECF (mmol/L)	4.8±0.2	2.1±0.3*	5.5±1.1^#^

Data are presented as mean with standard error of the number of experiments indicated in parenthesis. Animal groups were sham, TBI and TBI with pyruvate treatment

* ***P*** < 0.05, comparing other groups with sham

# ***P*** < 0.05, comparing pyruvate with TBI alone

### Pyruvate effects on serum lipid peroxidation in TBI

**Figure 1:**
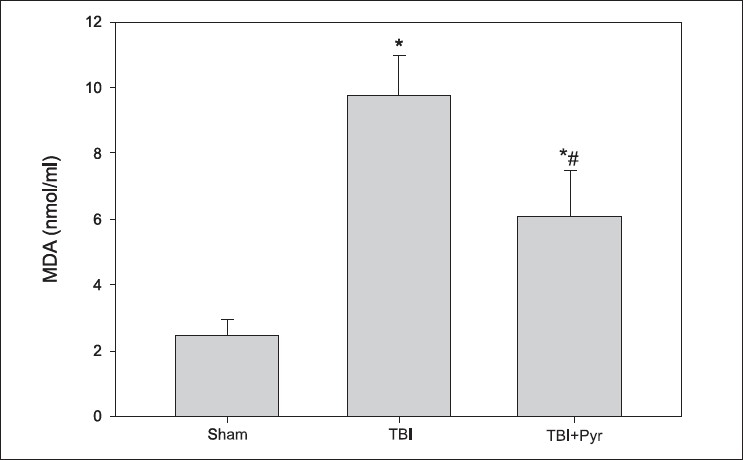
Effect of pyruvate on serum MDA levels in rats subjected to TBI. Data represent mean ± standard error of mean (n = 9 animals per group). *P < 0.05 compared to sham group #*P* < 0.05 compared to TBI group

Lipid peroxidation is a well established biomarker of oxidative stress in cells and tissues. Lipid peroxides are unstable and decompose to form complex series of compounds including MDA upon decomposition. As shown in Figure 1, there was a significant increase in serum MDA levels at 72 h post injury (approximately 5 fold) compared to sham values. Pyruvate treatment was significantly effective in minimizing the amount of lipid peroxidation, which was two fold higher than sham (P< 0.05).

### Pyruvate effects on total PDH enzyme levels in blood and brain

**Figure 2:**
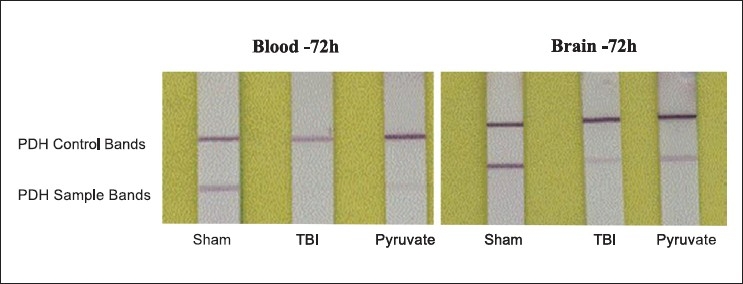
Representative figure of total PDH enzyme determined by dipstick test in blood and brain of rats with TBI. Dipsticks are of white color stripes and were placed on a paper with yellow background to increase the contrast. On left side, data of PDH protein bands are from blood samples collected at 72 h. Brain PDH levels were determined at 72-h post-injury periods. Note that in comparison to sham, amount of PDH was significantly reduced in TBI controls and pyruvate prevented its loss, although less visible in blood than brain tissue. These results are consistent with the lactate/pyruvate ratio and glucose levels in blood samples at 72 h

Figure 2 depicts that in TBI controls, total PDH enzyme in blood was significantly reduced in comparison to sham and pyruvate treatment was significantly effective in preventing the loss of PDH following TBI - as indicated by a faint protein band. The intensity of PDH bands in blood was 1.7 ± 0.2, 0.2 ± 0.03 and 0.7 ± 0.02 in sham, TBI and TBI + pyruvate groups (M ± SE, *n* = 6 animals per group). Also, in hippocampus from the same animals, TBI significantly reduced the total PDH and pyruvate administration prevented its loss. In addition, total PDH measured in brain by dipstick test (Intensity- 2.2 ±0.1, 0.3 ± 0.03, 0.9 ± 0.1 in sham, TBI and TBI+pyruvate)was significantly higher than the blood, P< 0.05. PDH activity is mainly regulated by the change in redox status such as lactate/pyruvate ratio, which was seen in our experiments [Table 1]. If this is true, then the measurement of systemic PDH contents using Dipstick test should be a reliable indicator of the severity of brain injury following TBI.

### Pyruvate effects on GFAP and PDH E1-∞ subunit expression in TBI brain

**Figure 3:**
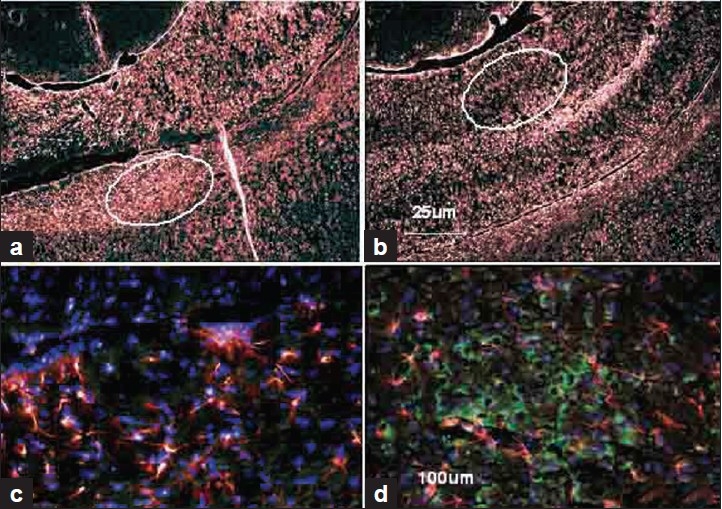
Effect of pyruvate treatment on PDH E1-∞ and GFAP contents in the right hippocampus of a rat. Fig 3a and 3b are at lower magnification (25×), while 3c and 3d (circled in a and b) at the higher magnification (100×). 3a and 3c are brain injuries without pyruvate. b and d are with pyruvate. Brain injury marker of gliosis . GFAP (red) was mainly localized around the injury in star-shaped branched astrocytes. PDH (green) was seen in neuronal bodies. 3a and 3c are showing very little PDH (green) and more red (GFAP) in comparison to pyruvate-treated TBI animals in 3b and 3d

A representative photomicrograph of right hippocampus of a rat immunostained for PDH E1-∞ and GFAP antibodies [Figure 3]. Pyruvate treatment of rats subjected to TBI for 72 h caused significant stimulation of PDH E1-∞ and less GFAP expression (marker of gliosis-brain injury) in comparison to the injured area with vehicle treatment [Figure 3a and 3b; lower magnification: 25×]. On higher magnification [100× – Figure 3c and 3d], GFAP (red) was mainly localized in star-shaped astrocytes, while PDH E1-∞ (green) was seen in neuronal bodies and not in filaments because mitochondria are mainly localized in neuronal bodies.

## DISCUSSION

In a fluid percussion type of closed head injury rat model, we have demonstrated the altered systemic metabolic responses to TBI in terms of reduced base excess, increased lactate-pyruvate ratio and elevated serum glucose levels. We have also shown that small alterations in total systemic PDH enzyme levels can influence the outcome of TBI, and pyruvate was significantly effective in reversing these harmful metabolic consequences by reducing gliosis and in preventing the loss of PDH enzyme. The PDH complex is one of the key mitochondrial matrix enzymes that catalyze the oxidative decarboxylation of pyruvate to acetyl CoA, nicotinamide adenine dinucleotide (the reduced form, NADH) and CO_2_. Pyruvate dehydrogenase is also an important link between aerobic and anaerobic cellular metabolic pathways and is very sensitive to oxidative stress in comparison to other mitochondrial enzymes.[18] The activity of PDH enzyme is mainly regulated by the phosphorylation and dephosphorylation of its sub unit E1-∞. In this study, we found a significant inhibition of E1-∞ in injured brain when compared with sham or pyruvate treated animals. The possible mechanisms by which pyruvate is effective in preventing the brain injury and loss of PDH activity may be 1) due to its antioxidant properties and 2) to optimize mitochondrial metabolism by providing substrate to PDH enzyme reaction and anaplerotic TCA cycle precursor (pyruvate carboxylase).

Astrocytes are the most common cell type in mammalian brain. Glial fibrillary acidic protein (GFAP) constitutes intermediate filaments (known also as nanofilaments) as part of the cytoskeleton in astrocytes. Reactive gliosis is a response of astrocytes to a variety of insults that is characterized by hypertrophy of the cell bodies and processes, and an increase in the expression of GFAP; the signal that regulates the transition to the reactive state. In the initial stages of brain injury, reactive astrocytes have a neuroprotective effect to enhance neuronal survival. In advanced stages of TBI, they have also been shown to inhibit CNS regeneration. In this study, TBI induced a significant degree of gliosis marked by increased GFAP and star-shaped astrocyte cell bodies indicating the presence of injured and dysfunctional astrocytes in the hippocampus. Also, PDH expression in the same area of hippocampus was significantly reduced suggesting a compromised metabolic pathway, and pyruvate was significantly effective in preventing the gliosis as well as in preventing the loss of PDH enzyme associated with TBI. In this study, we have examined only hippocampus area of the brain, because it is mainly related to the short term memory and focus, and these neurological functions are mainly affected by mild to moderate closed head injury.

The biochemical disturbances associated with TBI and neuronal cell death in the hippocampus mainly involves oxidative stress, such as excitotoxicity and membrane disruption due to lipid peroxidation. To the best of our knowledge, this is the first study to report the correlation of blood PDH contents, lipid peroxidation and severity of brain injury. The effect of lipid peroxidation on membrane lipids can markedly alter membrane functions such as intracellular homeostasis and transport mechanisms. Malondialdehyde is one of the several low-molecular-weight end products formed via the decomposition of certain primary and secondary lipid peroxidation products. At low pH and elevated temperature, MDA readily participates in nucleophilic addition reaction with 2-thiobarbituric acid (TBA), generating a red, fluorescent 1:2 MDA:TBA adduct. In our present study, serum MDA levels were significantly higher in TBI rats when compared with sham- or pyruvate-treated animals; this confirms the occurrence of systemic oxidative stress after brain injury. In addition, reduced serum MDA levels by pyruvate treatment confirm our previous findings about the antioxidant property of sodium pyruvate.[9,19,20] Although, the results of our dipstick assay test could confirm that TBI results in loss of total PDH enzyme in brain, which can have grave consequences on cell metabolism and neuronal survival, but PDH enzyme levels in whole blood were found to be significantly lower than the brain tissue. This variation may have resulted from 1) the effective dilution of the free radicals and other metabolic perturbations; as these agents cross the blood-brain barrier and enter into the systemic circulation, and 2) variations in the mitochondrial contents in 20 µl whole blood vs. 20 µg brain tissue protein. We believe that strong signals for total PDH enzyme, as well as for the active PDH could have been obtained in isolated PBMC, which was not possible in this study with small amount of blood in rats when compared with large animals such as pigs or in humans. In spite of these limitations, total PDH and its enzyme activity measured by dipsticks have many advantages over traditional spectrophotometric methods, such as fast response in 30 minutes, small size convenience, enzyme specificity, and no need of mitochondrial isolation. It can be performed in only 25-50-µl sample.

## CONCLUSIONS

The deficient energy metabolism and cell death in brain following TBI may be due to increased oxidative stress and loss of PDH activity in brain. The neuroprotective effects of pyruvate are mediated through its antioxidant mechanisms which can maintain global redox status, decrease lipid peroxidation and prevent the loss of PDH enzyme that occurs after TBI. The levels of PDH enzyme in blood can be measured using dipstick based immunoassay, and these values can predict the severity of brain injury in TBI. Key Messages: The levels of PDH enzyme in blood can predict the severity of brain injury and oxidative stress associated with head injury.
